# Tracing the active genetic diversity of *Microcystis* and *Microcystis* phage through a temporal survey of *Taihu*

**DOI:** 10.1371/journal.pone.0244482

**Published:** 2020-12-28

**Authors:** Helena L. Pound, Steven W. Wilhelm

**Affiliations:** Department of Microbiology, The University of Tennessee, Knoxville, Tennessee, United States of America; INRA, FRANCE

## Abstract

Harmful algal blooms are commonly thought to be dominated by a single genus, but they are not homogenous communities. Current approaches, both molecular and culture-based, often overlook fine-scale variations in community composition that can influence bloom dynamics. We combined homology-based searches (BLASTX) and phylogenetics to distinguish and quantify *Microcystis* host and phage members across a summer season during a 2014 *Microcystis-* dominated bloom that occurred in Lake Tai (*Taihu*), China. We found 47 different genotypes of the *Microcystis-*specific DNA-dependent RNA polymerase (*rpo*B), which included several morphospecies. *Microcystis flos-aquae* and *Microcystis wesenbergii* accounted for ~86% of total *Microcystis* transcripts, while the more commonly studied *Microcystis aeruginosa* only accounted for ~7%. *Microcystis* genotypes were classified into three temporal groups according to their expression patterns across the course of the bloom: early, constant and late. All *Microcystis* morphospecies were present in each group, indicating that expression patterns were likely dictated by competition driven by environmental factors, not phylogeny. We identified three primary *Microcystis*-infecting phages based on the viral terminase, including a novel *Siphoviridae* phage that may be capable of lysogeny. Within our dataset, *Myoviridae* phages consistent with those infecting *Microcystis* in a lytic manner were positively correlated to the early host genotypes, while the *Siphoviridae* phages were positively correlated to the late host genotypes, when the *Myoviridae* phages express putative genetic markers for lysogeny. The expression of genes in the microcystin-encoding *mcy* cassette was estimated using *mcyA*, which revealed 24 *Microcystis-*specific genotypes that were negatively correlated to the early host genotypes. Of all environmental factors measured, pH best described the temporal shift in the *Microcystis* community genotypic composition, promoting hypotheses regarding carbon concentration mechanisms and oxidative stress. Our work expounds on the complexity of HAB events, using a well-studied dataset to highlight the need for increased resolution of community dynamics.

## Introduction

Bodies of water around the world are blighted by annual blooms of algal biomass. Known as harmful algal blooms (HABs), these events of economic and environmental concerns are often associated with the production of toxic compounds and/or excessive biomass accumulation [1, 2]. Most bloom events, be they summer or winter / freshwater or marine, occur when a single genus of algae evades normal biological constraints and achieves numerical dominance in a community [3]. As such, events are typically referred to by the name of the dominant genus: for example, in China’s Lake Tai, (*Taihu* in Mandarin) these are generally referred to as *Microcystis* spp. blooms. The annual reoccurrence of similar species has been established in scientific literature, providing an excellent study system for genetic shifts in microbial communities [4].

Ecosystem processes are driven by the composition and function of the species present. Genotypic diversity of these species is of critical importance to community stability and performance [5]. This diversity can be catalogued in several ways, including by the richness and evenness of species or subspecies present and/or the number and composition of functional roles present [6, 7]. Genotypic diversity can arise in multiple ways, and is an ongoing process [8]. At the same time selective pressures are constant for microorganisms. Thus genetic drift occurs based on the stochastic effects of random selection [9], niche partitioning and resource utilization efficacy [10], environmental selection, and biotic interactions with fellow community members including predation [11] and symbiosis [12]. Composite genotypic diversity in an ecosystem can influence the phenotypic community, which governs many ecosystem traits including biomass accumulation, the production of secondary compounds, and resiliency to abiotic and biotic stressors.

Genetic diversity may become particularly important during HAB events, given the presumed lack of community diversity associated blooms. Although blooms are typically dominated by only a few species, commonly members of the same genus, it is critical to remember that these communities are not homogenous. Not only are there many different genotypes or strains of the dominant taxa present, the spatial and temporal distribution of these genotypes can vary widely over the bloom duration. Indeed, there have been many observations of genotypic shifts in *Microcystis* spp. blooms, even over the course of a single bloom event [13–16], where the focus is often on the ability (or lack there-of) of these cyanobacteria to produce to potent hepatotoxin microcystin (*aka* “fast-death factor”, [17]). Most studies have used polymerase chain reaction (PCR) approaches to characterize various genotypes and their toxicity [18], which comes with the risk of some genotypes being excluded. As toxicity is not unique trait of a single species or strain, the community composition of co-occurring toxic organisms should be considered in toxin research [19].

The democratization in molecular biological tools has provided many new avenues of study in HABs. However, these advanced tools can often overlook the constant disconnect that is the dissimilarities between results from lab studies and measured environmental processes. While controlled laboratory experiments and genomic sequencing of cultured isolates provide important information, they cannot represent the complexity of natural diversity and function present in an environment. For this reason, it may be less desirable to quantify the expression of genes in an environmental sample by recruiting sequences to the genome of a cultured isolate. This practice results in an underestimation of true diversity and can obscure species or strain level dynamics that may better reflect responses to environmental conditions.

Faced with the above, we sought to characterize the genotypic diversity of a spatially large and temporally extended *Microcystis* spp.-dominated bloom using metatranscriptomic sequencing. While previous efforts have examined specific subpopulations through recruitment to a model lab strain [4], nutrient cycling genes [20] and viruses as drivers of mortality [21, 22], our efforts here were focused on the specific question of how diverse the bloom forming community was. We used a homology-based BLASTX approach accompanied by phylogenetics to identify the species and strains of *Microcystis* that were present and to quantify how their distribution varied across the course of this bloom. The analyses revealed patterns in genotype-specific expression that were linked to abiotic and biotic environmental factors, including pH and viral infection, respectively. We were also able to link changes in genotypic-marker expression to changes in toxin gene activity. Our analyses provide evidence that diversity, as well as small-scale patterns, are much broader than previously thought within the *Microcystis* community and present new hypotheses regarding the role of genotypic diversity in bloom dynamics.

## Methods

### Sampling and sequencing

**Fig 1:**
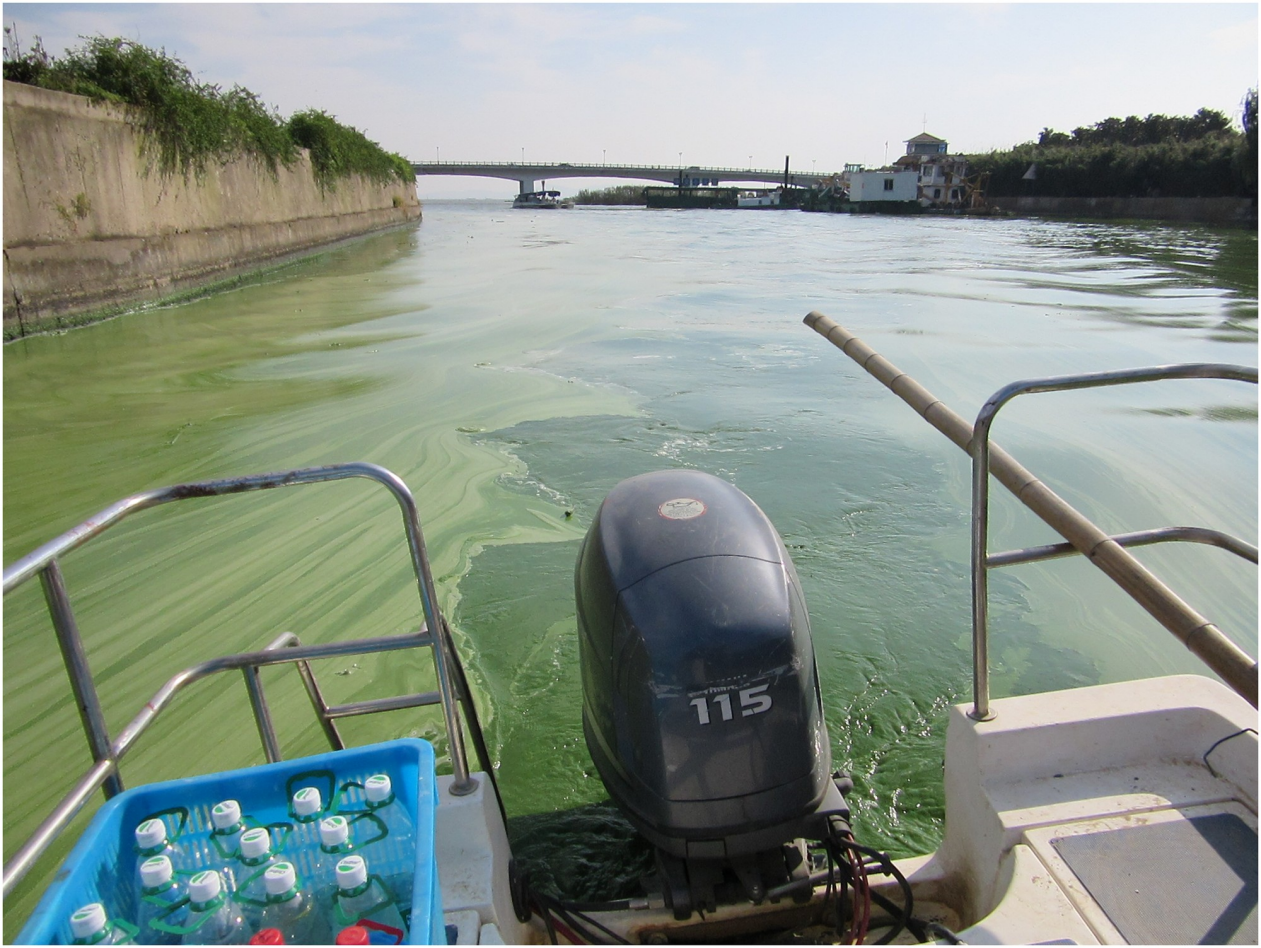
*Microcystis* spp. bloom. Image of the cyanobacterial community, dominated by *Microcystis* spp., from *Taihu*, China. Photo was taken during one of the sampling expeditions (October 7, 2014). Photo credit: SW Wilhelm.

Water samples were collected monthly from May to October from nine stations in Northwestern Lake Taihu in 2014 during daylight hours, as previously described [4, 20–22]. Based on anecdotal observations (*e*.*g*., see Fig 1), we know that a *Microcystis-*dominated bloom had established biomass by the time our sample collection started in June, yet continued to accumulate through to October. Whole water samples were collected at the surface and passed through a 0.22-μm pore-size Sterivex^™^ filter, excess water removed, and the unit filled with RNAlater^™^ (Invitrogen) until extraction. Basic physical parameters were also measured at time of sampling using a multiparameter water quality sonde (YSI 6600 V2) and include dissolved oxygen, water temperature, pH, turbidity, electrical conductivity, and phycocyanin. Total nitrogen (TN), total dissolved nitrogen (TDN), ammonium (NH_4_^+^), total phosphorus (TP), total dissolved phosphorus (TDP), orthophosphate (PO_4_^3–^), and chlorophyll *a* (Chl-*a*) were measured using standard methods (S1 Table). RNA extraction, sequencing: quality control details can be found in previous publications [4, 20–22]. A step by step protocol can be found at protocols.io describing the MoBio Powerwater DNA kit used to extract RNA and all modifications to the manufacturer protocol [23]. The resulting material paired-end sequences of 125 bp were generated at Hudson Alpha (Huntsville, AL, USA). Sequences are available from the MG-RAST database (“Lake_ *Taihu*_metatranscriptome_project”). Trimmed sequences from each sample were then combined to create a single assembly using MegaHit [22, 24].

### Host and virus detection

Genes of interest were identified and quantified using a hallmark gene approach previously described [25]. Briefly, our combined assembly was queried against a BLASTX v.2.6.0+ database containing hallmark protein sequences from isolated reference genomes. *Microcystis* host species were identified using the DNA-dependent RNA polymerase (rpoB) and *Microcystis* phage were identified using the phage terminase (S2 Table). The toxin encoding protein McyA was also used to characterize toxin production by *Microcystis* hosts and other cyanobacteria [26]. Sequences with BLASTX hits were considered candidates if they had an e-value of less than e^-30^ or e^-10^ (for hosts and viruses respectively), were greater than 300 bp, and were less than 95% similar to any other candidate [22].

### Host and virus taxonomy

**Fig 2:**
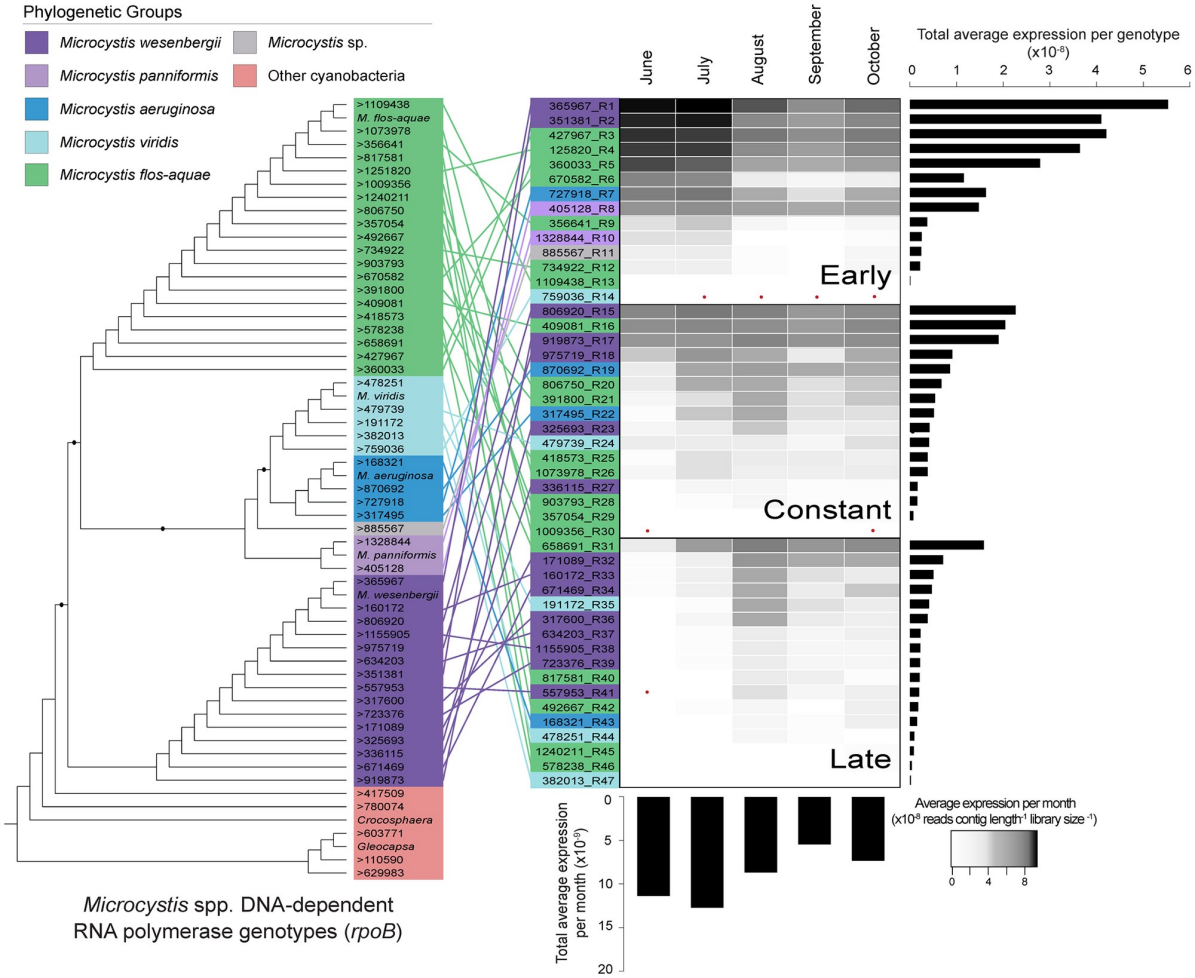
Expression of *Microcystis* genotypes. *Microcystis* DNA-dependent RNA polymerase (RpoB) cladogram of genotypes, colored by phylogenetic group. Genotypes then rearranged based on temporal phase, with heatmap of the average total expression of each genotype. Bar charts represent the average total expression for each genotype and each month. Red dots indicate no expression.

*Microcystis* host (RpoB and McyA) and virus candidate taxonomy was confirmed by placing the respective sequences on PhyML generated reference protein base phylogenetic trees using pplacer and visualized using iTOL v.4 [22, 27, 28]. All trees used to determine taxonomy are shown as cladograms to resolve taxonomy into distinct groups (Fig 2, S1 and S2 Figs). *Microcystis* spp. candidate sequences were first distinguished from other prokaryotes using a broad phylogenetic tree containing many prokaryotes and eukaryotes and then further characterized to species and genotype [22]. The *Microcystis* species-specific RpoB tree contained numerous isolates to increase genetic resolution. Approximate viral taxonomy was established by querying the candidates against the NCBI RefSeq non-redundant database, removing any false-positives or non-phage hits. The terminase tree for viruses contained many isolated viruses, both infecting and not infecting *Microcystis* and several virome sequences that originated from a *Microcystis* bloom and appeared in our BLASTX to the RefSeq database [29]. Within these, three groups were considered specific to *Microcystis*. The first contains the *Myoviridae* strains Ma-LMM01, MaMV-DC, and Node 34. Ma-LMM01 and MaMV-DC are lab isolates known to infect *Microcystis* hosts, and Node 34 is an uncultured phage sequence from a *Microcystis* bloom virome that is 99% similar to Ma-LMM01 and MaMV-DC [29–31]. The second group, referred to as Sipho I, is an unusual group with no isolated phage known to infect *Microcystis*. However, this group contains the *Siphoviridae* prophage-like sequence from *Microcystis aeruginosa* NIES-88 as well as Node 382, another uncultured phage from the Morimoto *et al*. virome [29]. Because these viral terminase sequences are 98.5% similar and found both in the environment and in a *Microcystis* host genome, we assume that this is a previously overlooked phage capable of infecting *Microcystis* hosts. Node 331 was not included in this group, as it is only 56% similar. The third group, referred to as Sipho II, contains the recently discovered *Siphoviridae* phage, Mic1 [32].

### Host and virus activity

Transcript activity was quantified by recruiting trimmed reads to candidate contigs that were trimmed to the length of the aligned hallmark gene and normalized to the trimmed contig length and library size [22, 33]. Monthly expression patterns were visualized using Heatmapper and the average expression of each genotype per month [34]. Genotypes were classified as early, constant, or late based on their patterns of average expression per month. Early genotypes displayed over 50% of their total expression during the months of June and July. Late genotypes displayed over 75% of their total expression during the months of August, September, and October. Constant genotypes did not have any month display higher than 25% of their total expression. The delineation between early and late months was originally proposed in Tang *et al*., based on changes in nutrient acquisition genes in the same dataset [4]. Pearson correlations between host and virus genotypes were established and visualized using RStudio. Correlations were corrected for multiple comparisons using the Benjamini Hochberg procedure [35]. Environmental drivers of host genotypic patterns were analyzed using canonical correspondence analysis (CCA) in RStudio.

### Novel virus detection

In our primary analysis, we noticed a high similarity between several phage transcripts (tail sheath, terminase, major capsid protein) and sequences including in a published *Microcystis* host genome, NIES-88 when performing BLASTX [36]. Intrigued, we ran the host genome through Phaster [37] and discovered a prophage-like portion. This portion of the genome was then annotated using OmicsBox v.1.2.4 (BioBam, Valencia, Spain) and visualized using CG View (S3 Fig) [38].

## Results

### The *Microcystis* community

Our species-specific *Microcystis* RpoB cladogram indicated that 47 of the 52 candidates previously identified by Pound *et al*. (2020) were *Microcystis* (Fig 2). The other five contigs originated from other cyanobacterial species. The most abundant (total reads) *Microcystis* species were *Microcystis flos-aquae* and *Microcystis wesenbergii*, with 43.5% and 42.4%, respectively, of the total *Microcystis* spp. expression. These species also had the highest number of genotypes, with 20 *M*. *flos-aquae* and 15 *M*. *wesenbergii*. Only four genotypes of *M*. *aeruginosa* were observed, and these accounted for only ~7.3% of the total *Microcystis* spp. expression. The classification of early, constant, and late genotypes varies in species composition, with no obvious delineation or temporal shift in species (Fig 2). The early genotypes account for 59.7% of the total expression, while the constant and late genotypes account for 27.2% and 13.2%, respectively. In parallel, a total of 30 McyA candidate contigs were identified: 24 most closely related to various *Microcystis* species, and six most closely related to other microcystin-producing cyanobacterial species (S1 Fig).

### *Microcystis*-infecting viruses

Our method yielded 52 phage terminase candidates, 15 of which were determined to be *Microcystis* specific (S2 Fig). Seven genotypes were most closely related to the well-documented *Myoviridae* phages, Ma-LMM01 and Ma-MVDC [30, 31]. The *Myoviridae* group represented 22.4% of the total *Microcystis*-associated phage expression. Only one candidate was most closely related to the Sipho I group containing our proposed novel phage, representing only 1.5% of the total *Microcystis* phage expression. The Sipho II group, containing Mic1, was the most abundant, with seven genotypes accounting for 76.1% of the total *Microcystis* phage expression. However, most of that expression came from just two genotypes and occurred in October.

### Seasonal shifts in expression

**Fig 3:**
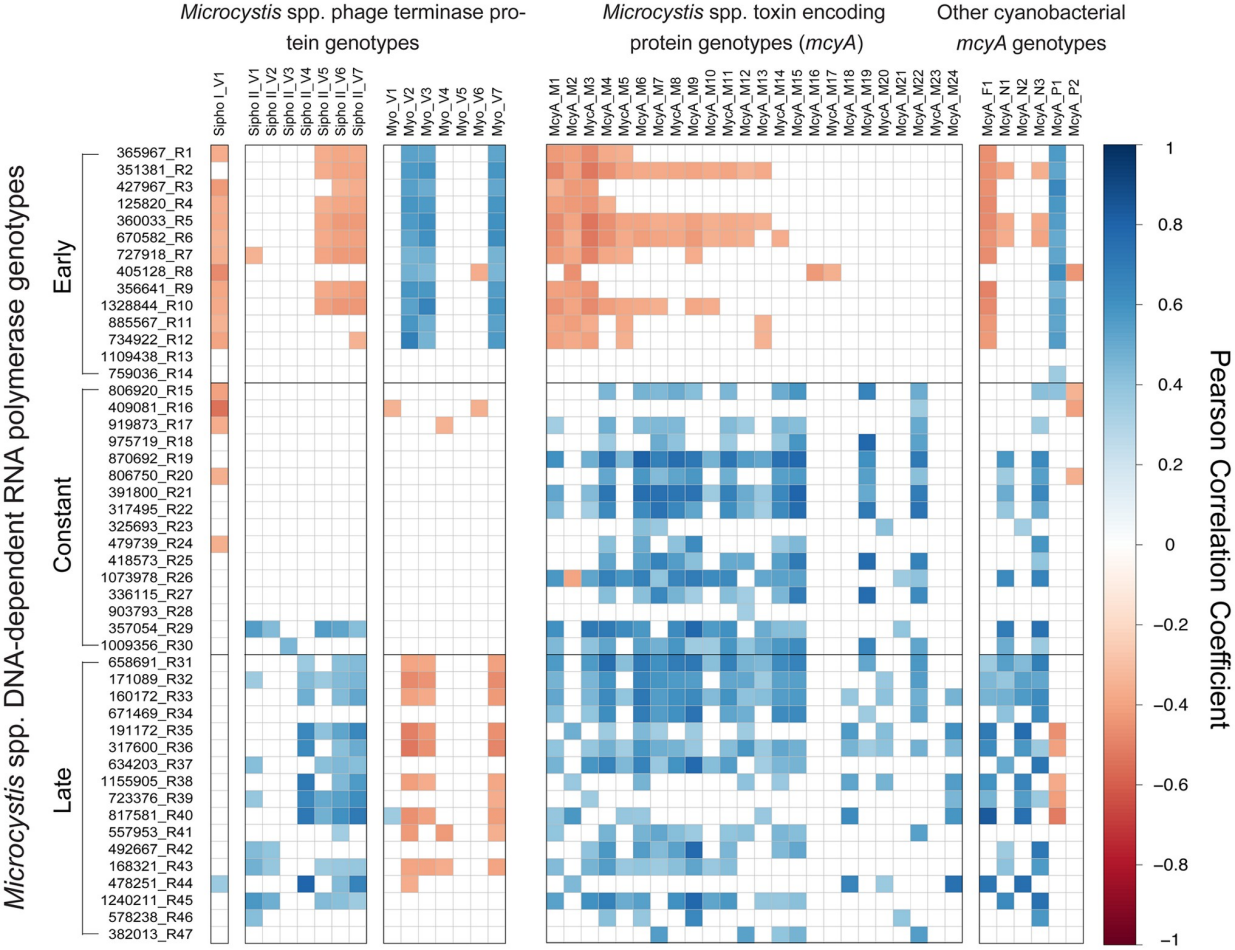
Host and phage correlation analysis. Pearson correlation analysis heatmap of *Microcystis* DNA-dependent RNA polymerase (*rpoB*) genotypes correlated to *Microcystis* phage terminase genotypes and *Microcystis* toxin genotypes. Blue indicates a significant positive correlation between expression values and red indicates a significant negative correlation between expression values. White indicates no significant correlations.

Pearson correlations indicated distinct seasonal patterns in expression of *Microcystis*infecting phage as well as toxin production genes (Fig 3). Early *Microcystis rpoB* genotypes show a positive correlation to the *Myoviridae* phage group, but negative correlations to both *Siphoviridae* groups. The inverse is true in the late genotypes, when *rpoB* expression is negatively correlated to the *Myoviridae* group and positively correlated to both *Siphoviridae* groups. Correlation patterns with phage in the constantly expressed genotypes are less defined but show an inverse correlation to the Sipho I group and no significant correlation to the Sipho II group.

Correlation patterns of *Microcystis rpoB* and *mcyA* are similarly delineated by early, constant, and late *rpoB* genotypes (Fig 3). Early *rpoB* genotypes showed a negative relationship to toxin-encoding gene expression, while constant and late genotypes are positively correlated with toxin-encoding gene expression. *mcyA* expression is an average of 2.1x higher in the late months (August, September, and October) than it is in the early months, indicating that early genotypes display decreased expression of the microcystin encoding gene, while late genotypes display increased expression of the microcystin encoding gene.

### Environmental variables

**Fig 4:**
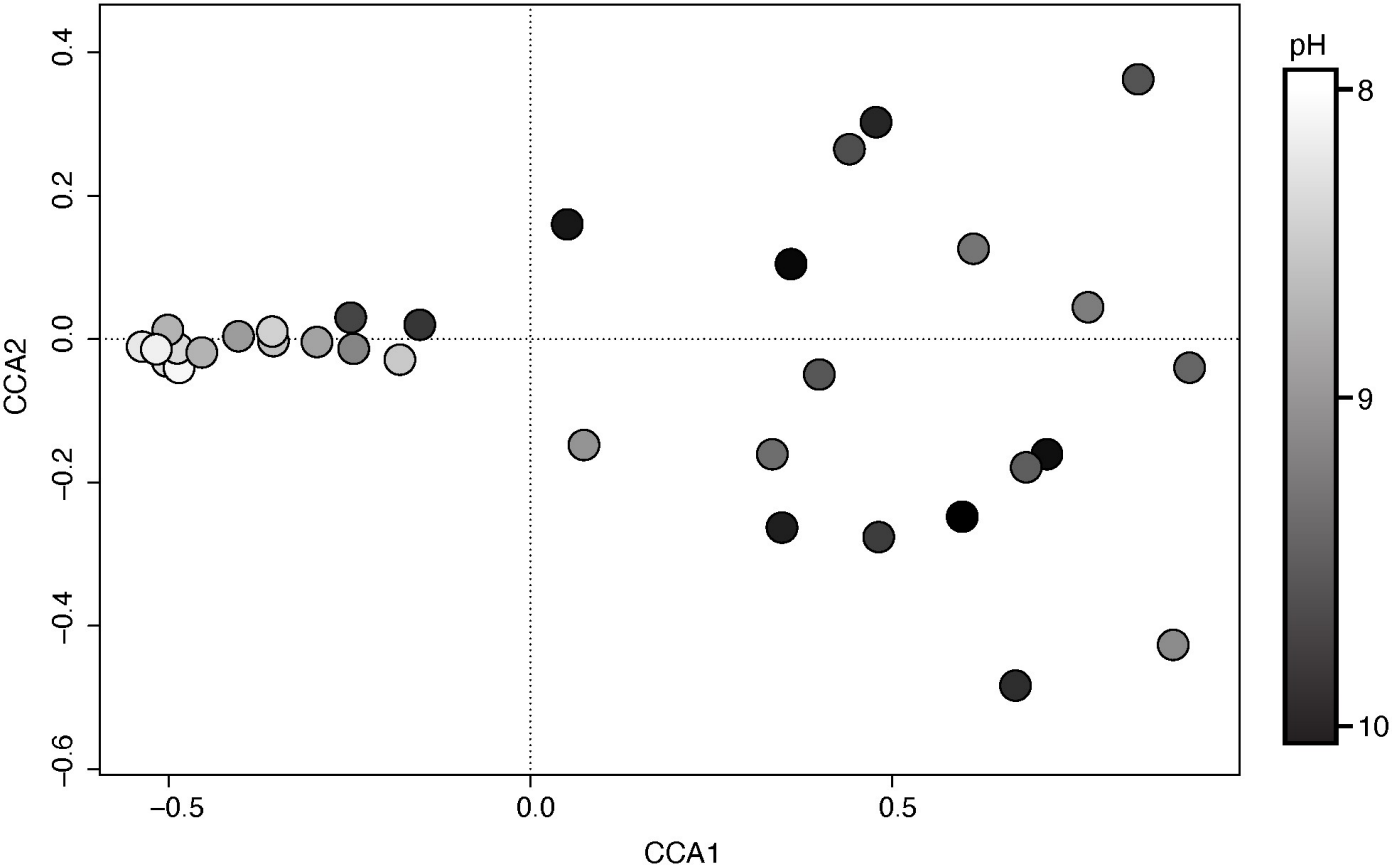
Canonical correspondence analysis. Canonical correspondence analysis of the proportional expression of all 47 *Microcystis* DNA-dependent RNA polymerase (*rpoB*) genotypes in each of our 33 samples. Color gradient indicates the pH of each sample.

To determine why *rpoB* genotypes have resolved into early, constant, and late groups, we used a canonical correspondence analysis to orient the proportional expression of all *rpoB* genotypes in each of our 33 samples. CCA1 described 66.2% of the variation in our samples, while CCA2 described 6.2% of the variation. Of the environmental variables measured, sample month and pH were the most positively associated variables to CCA1 (Fig 4) (S1 Table). Electrical conductivity, salinity, and total dissolved solids were also strongly associated with CCA1, in a negative direction. Total dissolved nitrogen and ammonium concentrations were the most highly correlated variables to the secondary axis, CCA2.

## Discussion

Cyanobacterial harmful algal blooms are a growing concern around the globe, and thus how they manifest–both in terms of function(s) and who is carry out these functions (*i*.*e*., which strains or species) is of critical importance to their management. The growing availability of genomic sequences has created opportunities for rapid assessment of environmental genomes and transcriptomes using approaches that involve “recruitment” (*i*.*e*., mapping of the unknown environmental sequences to well-studied lab isolates). Yet, while these approaches have been valuable to date and taught us much about how blooms are constrained, they are dependent on the choice of microbial strain for comparison. To move beyond these limits, we have developed a workflow to characterize the subtle variations in diversity that are commonly overlooked within genera that cause these events. Given that lab strains of *Microcystis* represent a broad spectrum of genetic potentials that could skew observations in the above approach, our approach creates an opportunity to more broadly capture spatial and temporal variation in *Microcystis* cell function *in situ* as multiple morphotypes and species are captured. When paired with parallel analyses of active viral infection of cells and toxin gene expression, a picture emerges that better resolves the variability that occurs in nature. The genotypic diversity present in Lake Tai reveals important features of *Microcystis* bloom dynamics in this system. Our findings suggest that the dominant species in the bloom are *M*. *flos-aquae* and *M*. *wesenbergii*, not *M*. *aeruginosa*. Our observations confirm other studies which employed PCR-based approaches to characterize toxic communities [39–41] in concluding that *M*. *flos-aquae* and *M*. *wesenbergii* were common in Lake Tai. Our observations further show a relationship between the distribution of subsets of these strains, transcription of toxin-encoding genes in the *mcy* cassette and active infections by dsDNA-phage thought to target *Microcystis*. In examining these data, we demonstrate how environmental conditions—in this case pH—may play a role in promoting or constraining the interactions between bloom success, viral infection and the production of a potent hepatotoxin. Taken together our observation begin to shed light on the complex interactions that result in the proliferation of a different genera with a large cyanobacterial bloom.

Much knowledge in microbiology stems from the use of lab cultures to mimic conditions found in the environment. To date, most studies in both the lab and natural systems have been performed with *M*. *aeruginosa* due to the broad availability of isolates as well as its implicit role as a major cyanotoxin producer *in situ*. However, our data suggest these strains may not provide the most accurate representation of bloom biomass in Lake Tai, nor bloom response to perturbations [42–44], as *M*. *aeruginosa* are only a subset (~ 7.3% of *Microcystis* spp. expression) of that community. *M*. *aeruginosa* NIES-843 was the first *Microcystis* species to have its genome fully-sequenced and closed [45], and therefore it has been the most widely applied genomic model for *Microcystis* blooms. As molecular biology becomes more broadly applied in natural systems, it is important to remember that an isolated reference organism may misrepresent the true diversity/function in a system. As our approach revealed 47 genotypes including at least 5 separate species of *Microcystis*, this work shows that going forward the use of a single reference genome could obscure the different patterns of seasonal expression we observed across genotypes.

Our analyses revealed that the *Microcystis* genotypes demonstrated different patterns of expression over the course of the bloom. There was a strict delineation between early and late genotypes, with additional genotypes occurring throughout the course of our sampling. These phases were initially characterized by Tang *et al*. (2018) as bloom “formation” and “maintenance”. They suggested these temporal groups were associated with changes in nutrient utilization strategies, based on temporal changes in the expression of nitrogen and phosphorus transport genes that matched the temporal patterns we observed in genotype expression within the same dataset [4]. From our observations, we now believe that the changes observed in nutrient utilization may have been, in part, a byproduct of the shifting genotypic composition. Unfortunately, it is impossible to separate cause and effect and it has become clear in recent years that nutrient concentrations measured in conjunction with biological parameters are as much the residual (as opposed to the cause) of the biology that is present [46]. We note that the temporal shift in genotypic populations does not indicate a morphospecies succession pattern. All three temporal groups are comprised of multiple *Microcystis* species, with no definitive dominance of any particular morphospecies in a given temporal group (Fig 2). This suggests that the various species present might occupy parallel, yet different functional niches within the ecosystem, allowing for co-occurring species groups [6]. This further suggests that attributions of functions to morphospecies may be inexact.

In addition to the 47 *Microcystis* genotypes we observed, we also noted a wide diversity of *Microcystis* phages. In a parallel to efforts to recruit to *Microcystis* genomes, most literature pertaining to *Microcystis* phages refer only two isolated *Myoviridae* phages, Ma-LMM01 and MaMV-DC [21, 30, 31, 47] and uses these as recruitment models. In the current study, our approach allowed us to detect these well-characterized *Myoviridae* phages, the newly characterized *Siphoviridae* phage, Mic1 [32], as well as a new *Siphoviridae* phage that we are confident infects *Microcystis* spp. This latter phage was originally “discovered” in our analyses of a genomic DNA scaffold of *M*. *aeruginosa* NIES-88, suggesting that it might have lysogenic potential (S3 Fig). Indeed, the genetic complement of this virus includes an integrase gene, which has not been observed in other *Microcystis* phages, although many have suggested the potential for lysogeny in *Microcystis* bloom systems [21, 48]. This NIES-88 phage portion does not appear to be a complete phage, indicating that it is likely remnant material. The presence of this phage in the host genome promotes questions regarding the role of these viruses in horizontal gene transfer and the rate at which phage and host genomes exchange material: there is a definite precedent for this being a common occurrence in filamentous cyanobacteria [49].

Just as the gene expression of our host genotypes resolved into three temporal groups, our three main classes of *Microcystis* phages did the same. The early phase of the bloom (June and July) was characterized by and over-representation of the *Myoviridae* phage transcripts, which had been observed previously [21]. Stough *et al*. used a single reference genome as bait to identify phage activity, yet in our effort we have shown that there were 7 genotypes present. The presence of multiple *Myoviridae* genotypes has been observed before *via* real-time PCR and might suggest rapid co-evolution with the host, or standing diversity [50]. While Ma-LMM01 and MaMV-DC were both originally isolated on *M*. *aeruginosa*, recent analysis of MaMV-DC indicates that it can limit the growth of *M*. *wesenbergii* and *M*. *flos-aquae*, although it did not form plaques [51]. This suggests that the *Myoviridae* genotypes we observed may infect several *Microcystis* strains */* species that were prominent in the spring. When the *Myoviridae* is highly expressed with the early host genotypes, infection by the two *Siphoviridae* groups are nearly absent. In fact, the single Sipho I genotype was not expressed at all during June or July, and four of the seven Sipho II genotypes were not expressed at all during June. Sipho II was much more highly correlated to the late host genotypes, when there was a decrease in lytic *Myoviridae* expression. This shift could be explained in a number of ways. The genotypic shift in hosts may reflect a shift in host susceptibility or viral specificity [50] or a successional overturn, either through density-dependent infection or lysogenic reproduction [21, 52]. However, it is important to note that Stough *et al*. observed an increase in genes associated with myocyanophage-lysogeny during the later months, when the late genotypes are expressed [21]. It is possible that the *Siphoviridae* phages only become active once the *Myoviridae* phage have shifted into a lysogenic reproductive cycle. Or, it might be that the shift to a lysogeny for the *Myoviridae* might prevent superinfection, causing a decrease in the expression of lytic *Myoviridae*. In any case, it is unclear whether the host or the viruses are driving the temporal patterns we observe.

The early host genotypes that strongly correlated to *Myoviridae* expression also strongly correlated (negatively) to the expression of the toxin coding *mcy*A gene. The constant and late genotypes are positively correlated with the expression of the toxin gene, even though actual toxin levels seem to decrease at the warmer temperatures typical of September and October [53]. Many studies have attempted to characterize genotypes and how toxin genes change in copy abundance over the course of a bloom [14, 15, 54]. Expression of *mcyA* occurs throughout the bloom, although there is an increase in the later months, providing a positive correlation to both the constant and late genotypes (S1 Fig). We note that again there is a disconnect between phylogeny and temporal patterns, given the diverse composition of *mcyA* taxonomic groups. Microcystin production is thought to rarely result from a single species or strain, but likely is a product of many co-occurring organisms [14, 16, 19]. Indeed, Otten and Paerl warn against using *Microcystis* species type to estimate toxicity, because the co-occurring presence of many species of various toxicities can obscure toxicity associations with particular species [40]. Even the expression of the *mcyA* toxin-encoding gene (or any gene in that cassette) is not a guarantee that an organism is capable or actively producing microcystin, as often other components of the cassette can be missing [55].

Although toxin gene expression and viral infection show similar temporal patterns, this does not suggest that they are directly related or regulated. More than likely, both observations are a function of cellular processes responding to similar external perturbations. Given that taxonomy is not capable of describing host activity, viral infection, or toxin gene expression, it is logical to hypothesize that the environment must be selecting for the genotypes we observed during various bloom phases. While previous studies have proposed that time of day could influence phage expression levels, we did not observe any pattern associated with diel cycling [*vis a vis* 29]. Our analyses revealed that one the primary environmental factor associated with the temporal shift in host *rpoB* expression was pH (Fig 4). System pH during cyanobacterial blooms is thought to increase due to the consumption of water column dissolved carbon dioxide. In parallel with this, pH is thought to influence *Microcystis* spp. carbon concentrating mechanisms, with evidence that *Microcystis* spp. are able to maintain growth at higher pH [56, 57]. It is unclear how pH may be influencing toxicity or viral infection, although we hypothesize that linkages to photosynthesis, oxidative stress and nutrient acquisition are all potentially involved [56]. Electrical conductivity and salinity were also strongly associated with the temporal shift in host *rpoB* expression. These two environmental parameters are highly self-correlated, representing the presence of sodium ions in the water. Combined with the strong association of total dissolved solids, we posit that this represents the presence of external terrestrial loading or resuspension, a hypothesis previously suggested by Wilhelm *et al*. [58]. This could also help explain why we do not observe a strong association with our nutrient parameters such as nitrogen or phosphorus. They are likely being turned over quickly by the biological population, particularly during the early months of the productive bloom, whereas sodium ions would remain in the water column.

Overall, our observations have allowed us to identify and quantify the abundant host and viral genotypes present in a freshwater cyanobacterial bloom system across a temporal profile. A major observation here is that the “bloom” does not appear to be a single genotype of one organism, but at least 47 different phylotypes that come from multiple species of the same genus. Temporal shifts in active viral infection (including transitions in the type of virus) and the expression of a key gene for toxin production confirm that the metabolism of *Microcystis* is likely regulated by a complex interaction with environmental drivers [43] and that these drivers influence the way researchers interpret bloom dynamics [46]. Our study highlights the complexity of bloom systems, and how environmental factors can vary and relate to similar organisms in different ways.

## Supporting information

S1 Fig *McyA* cladogram. Cladogram of toxin-encoding candidate contigs (*mcyA*). Inner color ring indicates taxonomic group and outer heatmap rings indicate the average expression of each candidate per month. Black dots indicate bootstrap values greater than 0.5 and red dots indicate no expression.
(TIF)

S2 Fig Terminase cladogram. Cladogram of phage terminase candidate contigs. Inner color ring indicates *Microcystis* phage group and outer heatmap rings indicate the average expression of each candidate per month. Black dots indicate bootstrap values greater than 0.5 and red dots indicate no expression.
(TIF)

S3 Fig NIES-88 genome. Genome scaffold map of NIES-88 (Accession number NZ_JXYX010000002). Orange open reading frames indicate phage-like genes.
(TIFF)

S1 Table Environmental parameters. Table of environmental parameters measured with each sample collected and the associated biplot scores for the *RpoB* canonical correspondence analysis.
(XLSX)

S2 Table Genotype sequences. Sequences of RpoB, McyA, and terminase genotypes identified and analyzed.
(XLSX)
