# Supplementary figures and images for: Tracing the active genetic diversity of Microcystis and Microcystis phage through a temporal survey of Taihu
Source: PLoS One. 2020 Dec 28;15(12):e0244482. doi: 10.1371/journal.pone.0244482 (PMC7769430; doi:10.1371/journal.pone.0244482)

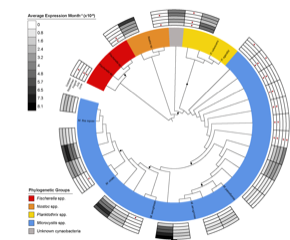

Supplement: S1 Fig — Cladogram of toxin-encoding candidate contigs (mcyA). Inner color ring indicates taxonomic group and outer heatmap rings indicate the average expression of each candidate per month. Black dots indicate bootstrap values greater than 0.5 and red dots indicate no expression. (TIF) [file pone.0244482.s001.tif]

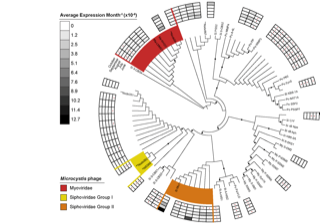

Supplement: S2 Fig — Cladogram of phage terminase candidate contigs. Inner color ring indicates Microcystis phage group and outer heatmap rings indicate the average expression of each candidate per month. Black dots indicate bootstrap values greater than 0.5 and red dots indicate no expression. (TIF) [file pone.0244482.s002.tif]

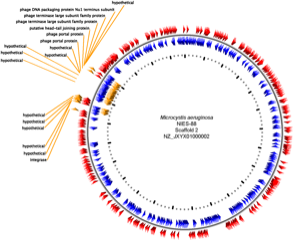

Supplement: S3 Fig — Genome scaffold map of NIES-88 (Accession number NZ_JXYX010000002). Orange open reading frames indicate phage-like genes. (TIFF) [file pone.0244482.s003.tiff]
